# Supplementary material for: Striated myocyte structural integrity: Automated analysis of sarcomeric z-discs
Source: PLoS Comput Biol. 2020 Mar 4;16(3):e1007676. doi: 10.1371/journal.pcbi.1007676 (PMC7075639; doi:10.1371/journal.pcbi.1007676)
Supplement: S1 Table — Column 1 is the stage of analysis at which a parameter is used, followed by its description in column 2. The third column is the value of the parameters used for analysis of the images, which had the resolution ~6 pixels/μm. (PDF) [file pcbi.1007676.s001.pdf]

Analysis Stage	Description	Value
Initialization	Pixel to micron conversion	6.22 pixels/µm
Diffusion Filtering	Diffusion time step	0.15 s
Diffusion Filtering	Standard deviation of Gaussian smoothing before calculation of the image Hessian	1 pixel
Diffusion Filtering	Standard deviation of Gaussian smoothing of the image Hessian	1.4 pixels
Diffusion Filtering	Total diffusion time; Sets number of iterations	1.5 s (11 iterations)
Top Hat Filtering	Radius of the flat disk-shaped structuring element used for the top hat filter	3 pixels (~0.5 µm)
Background Removal	Size of blocks to break image into	15 pixels (~2.5 µm)
Background Removal	Size of blocks considered noise in the condensed image	8 pixels (~25 µm)
Background Removal	Standard deviation of Gaussian smoothing to perform on image	1 pixel
Binarization	Size of small objects to be removed in the binarized image	8 pixels^2 (0.2 µm^2)
Skeletonization	Minimum branch size to be included in analysis	4 pixels
Actin Orientation Calculation	Sigma of the Gaussian weighting used to sum the gradient moments	3 pixels
Actin Orientation Calculation	Sigma of the derivative of Gaussian used to compute image gradients	1 pixel
Actin Orientation Calculation	Size of Gaussian filter kernel to perform on actin image	25 pixels
Actin Orientation Calculation	Sigma of the Gaussian used to smooth the final orientation vector field	3 pixels
Actin Orientation Calculation	Minimum reliability of actin orientation vectors	0.5
Actin Orientation Calculation	Standard deviation of Gaussian smoothing to perform on actin image	3 pixels
Actin Guided Segmentation	Minimum angle between α-actinin and local actin orientation for pixels to be considered perpendicular	0.7 ($>$~45°)
Actin Guided Segmentation	Size of local actin orientation	30 pixels (~5 µm)
Continuous Z-line Length	Maximum angle between pixels to be considered parallel and therefore continuous	0.9 ($<$~25°)
