# Supplementary figures and images for: Striated myocyte structural integrity: Automated analysis of sarcomeric z-discs
Source: PLoS Comput Biol. 2020 Mar 4;16(3):e1007676. doi: 10.1371/journal.pcbi.1007676 (PMC7075639; doi:10.1371/journal.pcbi.1007676)

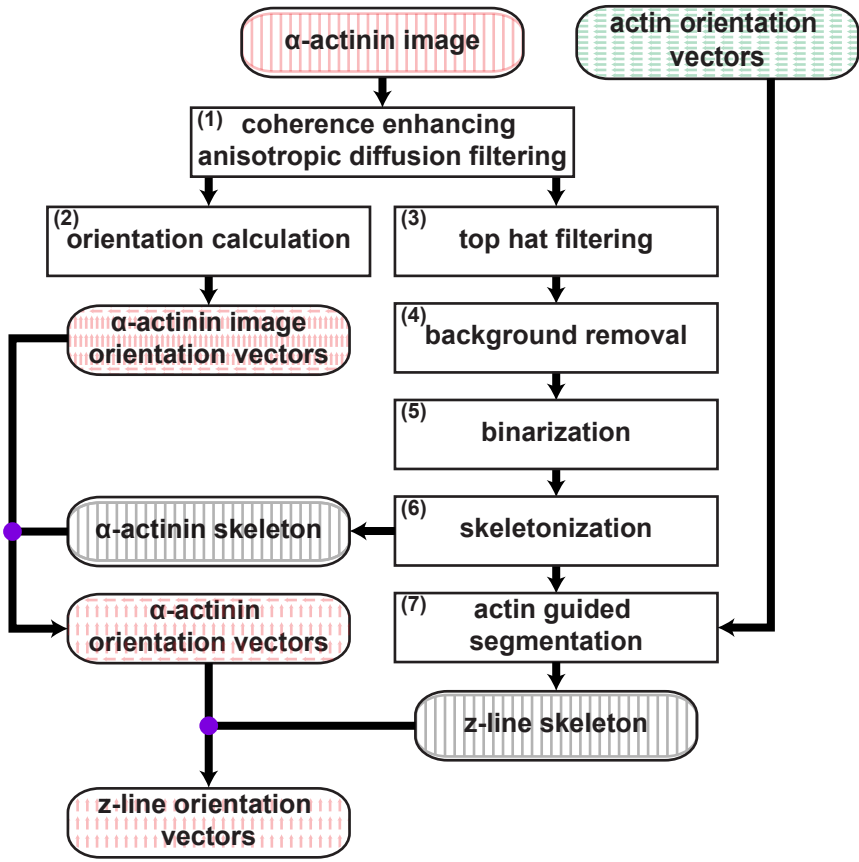

Supplement: S1 Fig — Squared boxed text indicates an image analysis step in ZlineDetection. Rounded boxed text indicates an image or matrix, where binary skeletons are shaded gray, matrices containing information about α-actinin stained images are shaded red, and matrices containing information about actin stained images are shaded green. Purple circles indicate matrix multiplication. On a computer with 32 GB of RAM, ZlineDetection took ~30 s to analyze a 1024 x 1344 image. (PDF) [file pcbi.1007676.s002.pdf]

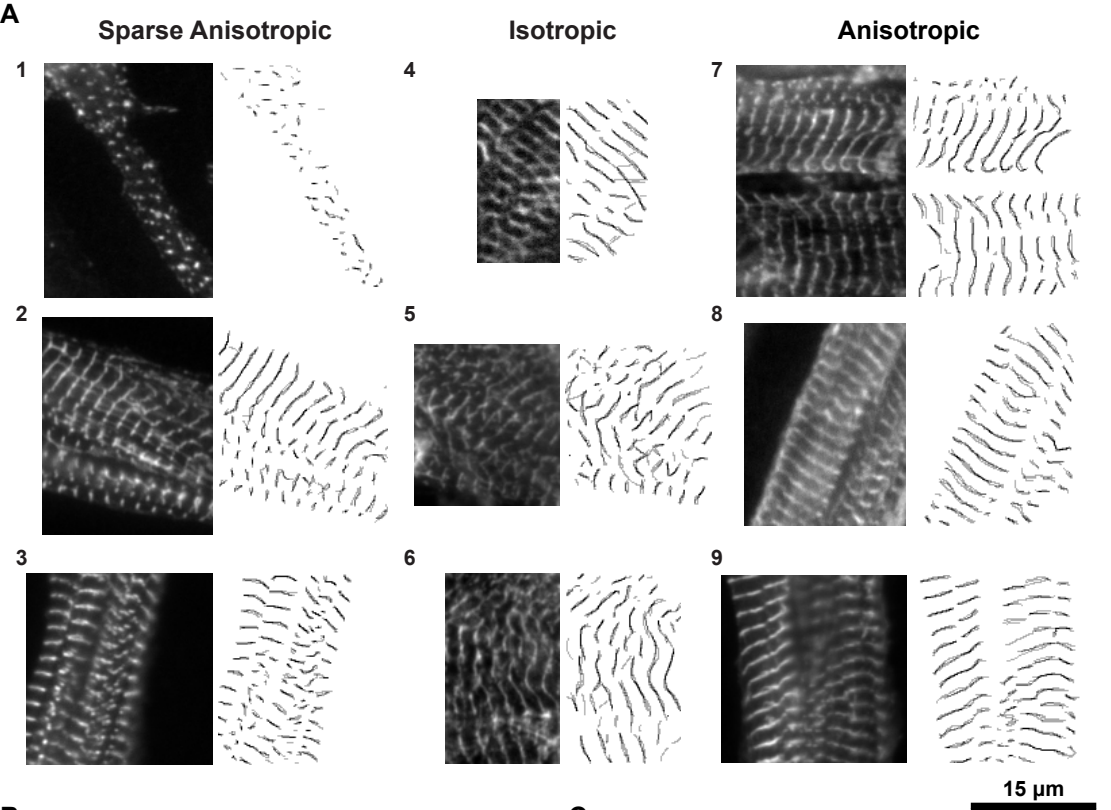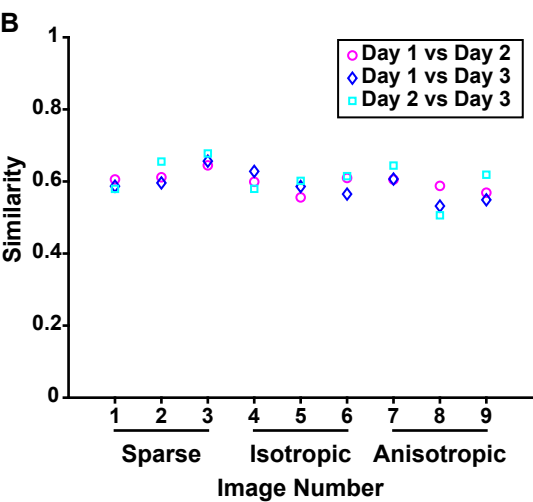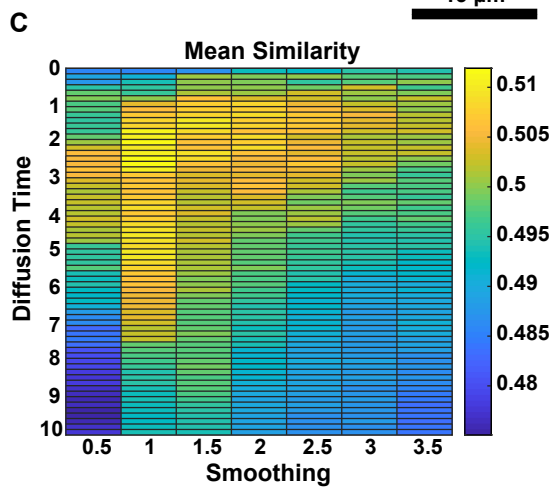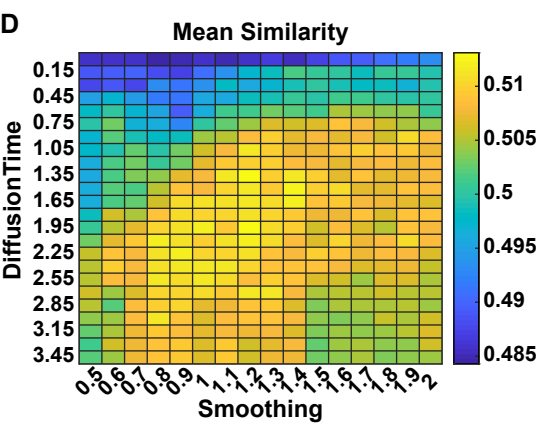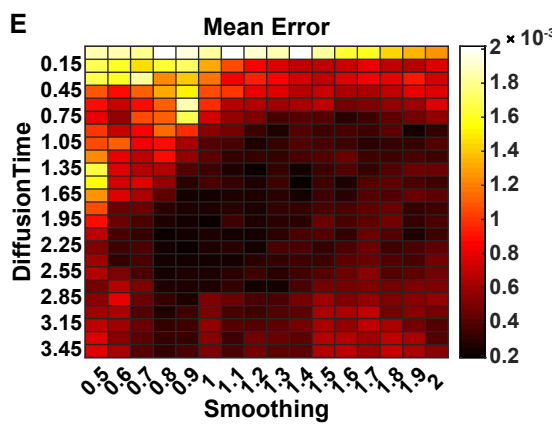

Supplement: S2 Fig — A, Sections of anisotropic, isotropic, and sparse anisotropic tissues with manually traced z-lines, which was done three times. B, Similarity between the three different manual traces of z-lines. C, Average similarity for each set of diffusion filtering parameters. D, Average similarity for a more refined range of diffusion filtering parameters. E, Error for refined range of diffusion filtering parameters (Eq 1). (PDF) [file pcbi.1007676.s003.pdf]

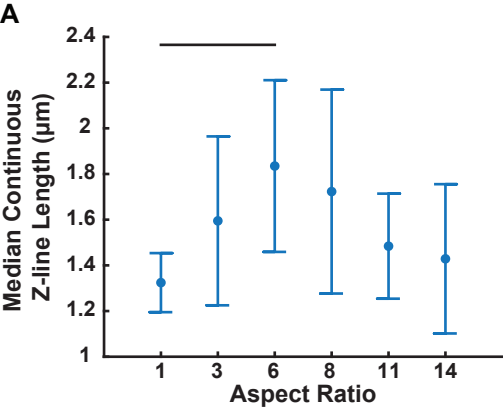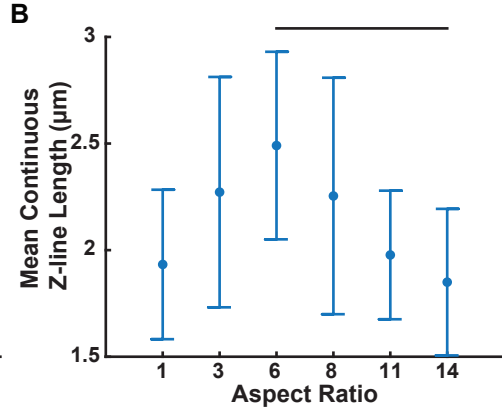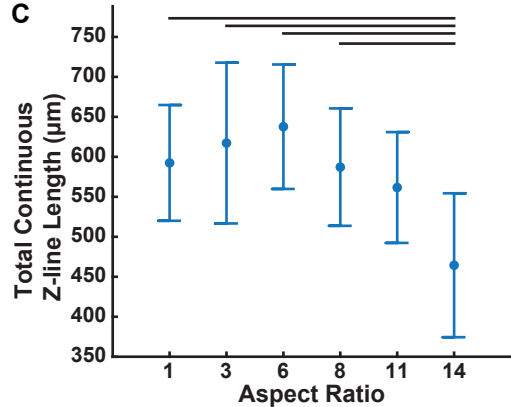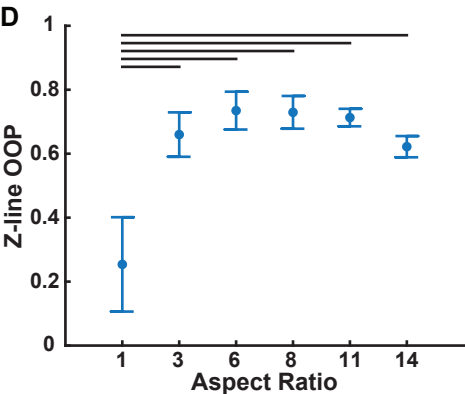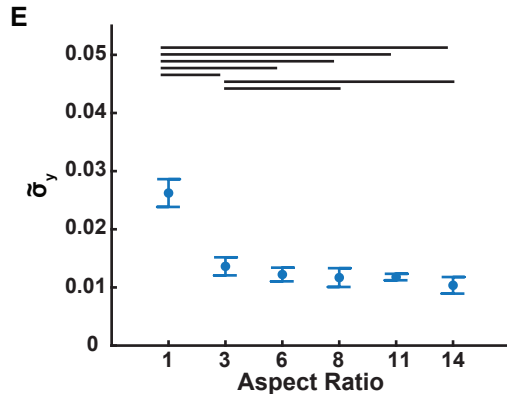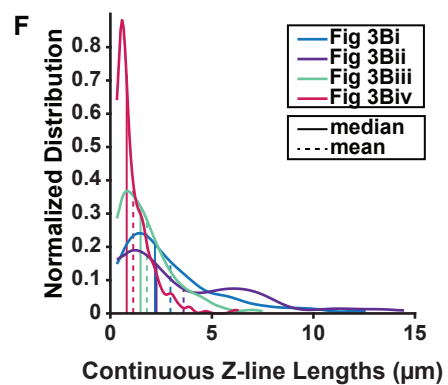

Supplement: S3 Fig — For the good cells of each aspect ratio, the mean and standard deviation are shown for the median continuous z-line length (A), mean continuous z-line length (B), total continuous z-line length (C), z-line OOP (D), and the estimated force along the axis perpendicular to principle axis (E). F, Normalized distribution of continuous z-line lengths for the representative cells in Fig 3. Groups were compared using ANOVA with Tukey’s test p <0.05 (black horizontal bars above data). (PDF) [file pcbi.1007676.s004.pdf]

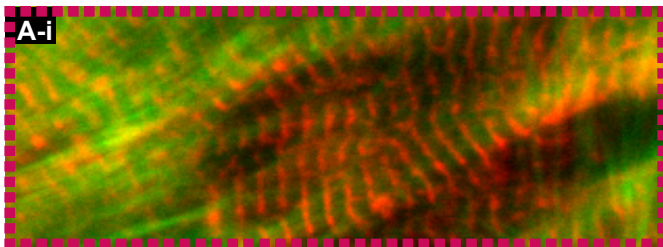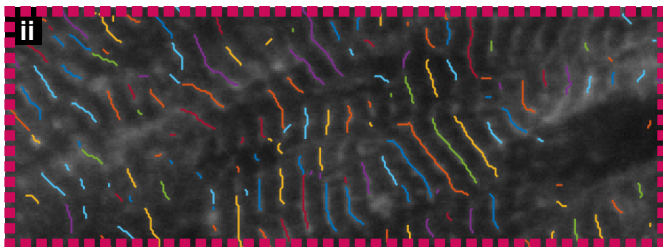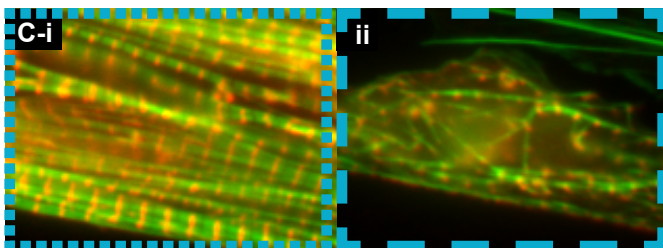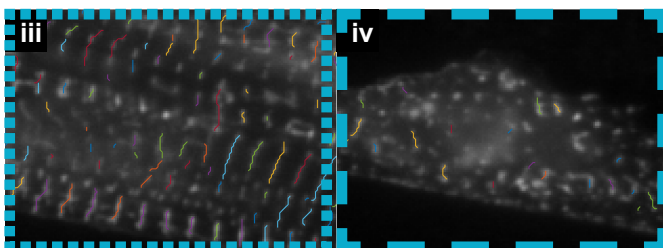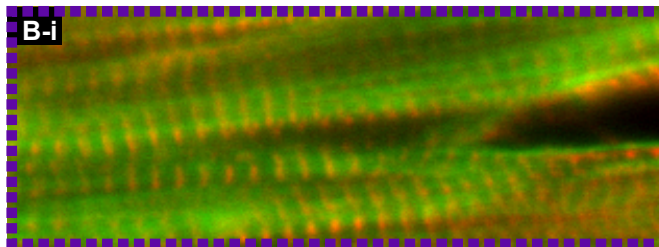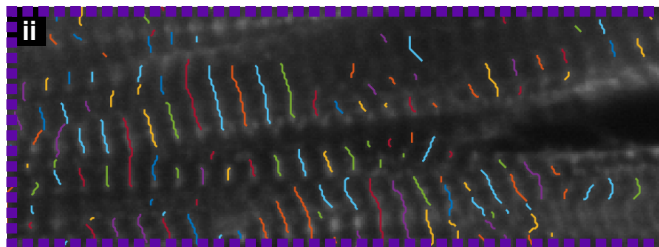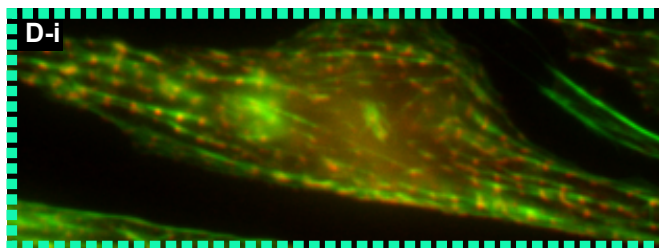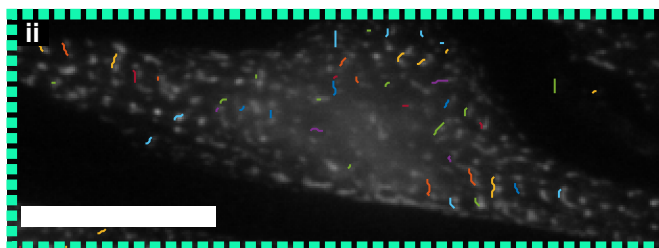

Supplement: S4 Fig — Sections of cardiac tissue shown in Fig 4A–4D stained for actin (green) and α-actinin (red) on a uniform layer of FN (Ai), FN in lines (Bi), FN in lines with sparsely seeded cardiomyocytes (Ci), and FN in lines with cardiomyocytes treated with BDM (Di) and their corresponding continuous z-lines (A-Dii). Scale bar: 15 μm. (PDF) [file pcbi.1007676.s005.pdf]

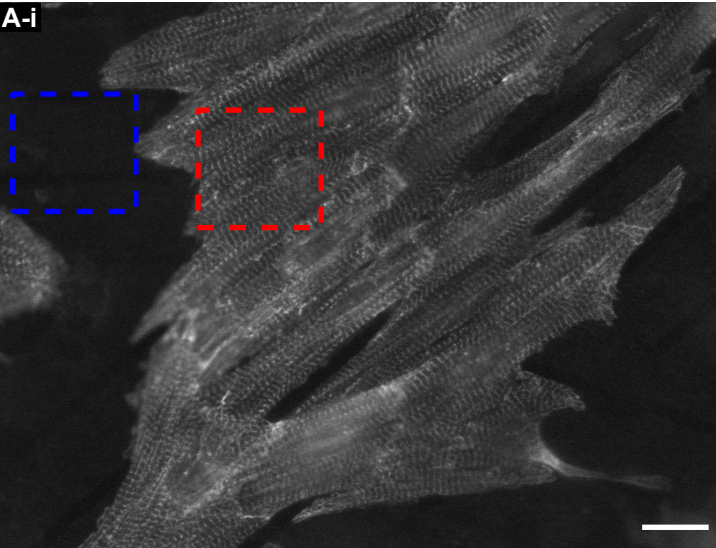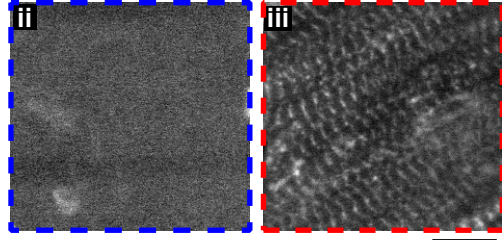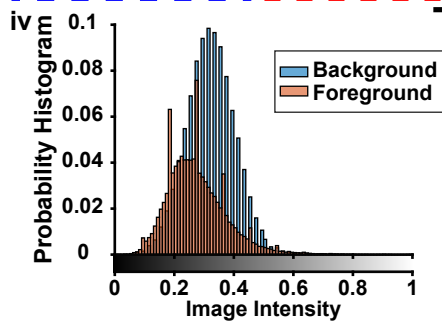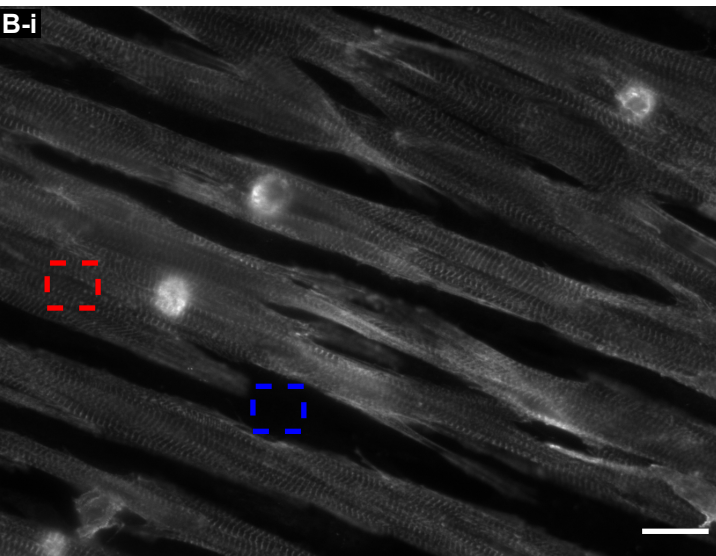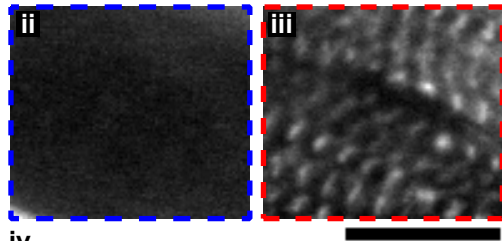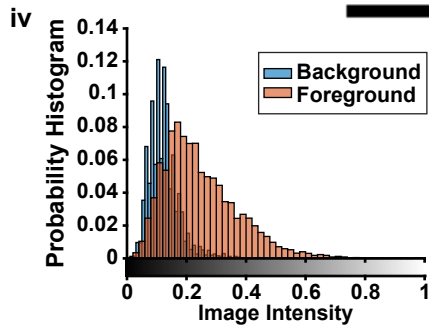

Supplement: S5 Fig — A, α-actinin stained image of poor imaging quality. B, Example of good imaging quality. For both A and B, the background (i), foreground (ii), and distribution of intensities (iii) are shown. Scale bars: (A-B i) 20 μm; (A-B ii-iii) 10 μm. (PDF) [file pcbi.1007676.s006.pdf]

**A**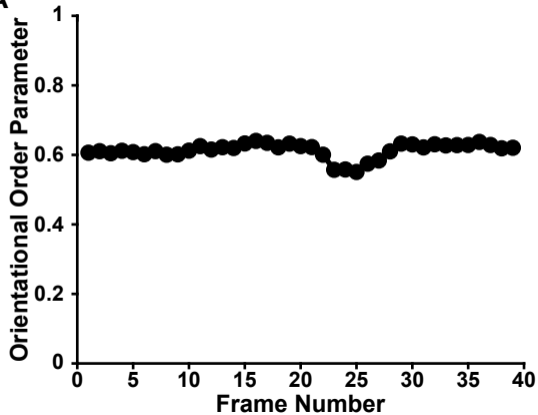**B**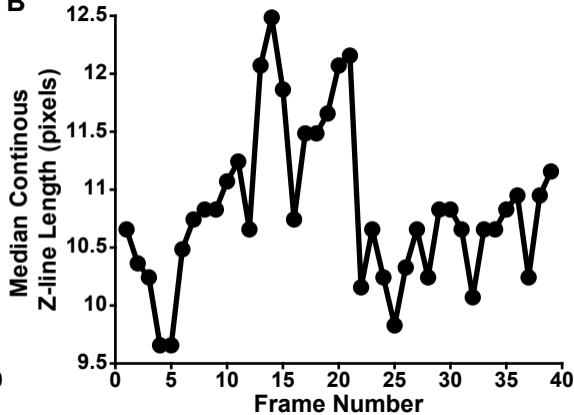

Supplement: S6 Fig — Results of analyzing titin-GFP sarcomere reporter human induced pluripotent stem cell-derived cardiomyocyte published by Sharma et al. [81]. (A) Orientational order parameter and (B) median continuous z-line length in pixels as a function of frame number. As expected, the OOP was relatively constant throughout the contraction, while the median continuous z-line length varied due to non-synchronous contractions of neighboring myofibrils. (PDF) [file pcbi.1007676.s007.pdf]
