# Supplementary material for: Striated myocyte structural integrity: Automated analysis of sarcomeric z-discs
Source: PLoS Comput Biol. 2020 Mar 4;16(3):e1007676. doi: 10.1371/journal.pcbi.1007676 (PMC7075639; doi:10.1371/journal.pcbi.1007676)
Supplement: Attachment — Submitted filename: ResponseReviewComments_20200117.pdf [file pcbi.1007676.s008.pdf]

Response to Reviewers
"Striated myocyte structural integrity: automated analysis of sarcomeric z-discs"

We thank the reviewers for their thoughtful comments, and we believe our revised manuscript has been greatly improved by addressing their concerns. Our responses are detailed below in regular font, while *the reviewer comments are in italics.* The added/changed text in the manuscript is highlighted, and the corresponding page and line numbers are given with the responses below.

Reviewer #1

> *The authors present a set of image analysis tools that enable quantification of z-line architecture in contracting myocytes, and use these to elucidated the mechanisms by which highly elongated cardiomyocytes lose efficiency in force production. Their code ZlineDetection evaluates sarcomere architecture in a way that mimics expert imaging analysis, and appears to be a general tool that enables this analysis to occur objectively. The methods are straightforward and robust. The examples shown demonstrate clear utility for the methods. The reviewer believes that the software meets a clear need in the field.*
>
> *The only disappointment with the paper was that the GitHub link did not work; the reviewer would have liked to have tried the software.*

We thank the reviewer for their time and their interest in trying `ZlineDetection`. We have made the Github repository public, and ZlineDetection can be accessed at the link provided in the manuscript (`https://github.com/Cardiovascular-Modeling-Laboratory/zlineDetection`) where the user guide can also be found (`https://github.com/Cardiovascular-Modeling-Laboratory/zlineDetection/blob/master/userGuide.pdf`).

Reviewer #2

> *The manuscript entitled 'Striated myocyte structural integrity: automated analysis of sarcomeric z-discs' describes a MatLab algorithm that can be used to assess the order of sarcomeres in striated muscle cells. This is an automated technique that uses z-disc and actin co-staining to reduce z-disc characterization error. The technique provides a set of parameters to determine characteristics of z-disc architecture and differences generated by experimental treatments. The technique will undoubtedly be of interest to the field, although not the only algorithm used to make these types of measures. Nevertheless, useful alongside existing technologies.*

We thank the reviewer for their questions and suggestions. We believe our manuscript has been immensely improved by including a comprehensive comparison between `ZlineDetection` and existing algorithms. Additionally, the inclusion of how `ZlineDetection` can be useful in analyzing stem cell-derived cardiomyocytes will make the benefit of our paper more evident to a wider audience.

> *Comments:*
> *How does this algorithm compare to that of for instance Sutcliffe et.al. (PMID: 29352247) and others? What are the pros and cons of using other techniques versus the one presented in this manuscript? Could one make a direct comparison between the techniques to highlight what this current algorithm achieves over previous methods?*

A key difference between between `ZlineDetection` and existing algorithms is the use of the actin stain to decouple multiple facets of z-line and myofibril architecture. A metric that is unique to `ZlineDetection` is the relative amount of α-actinin pixels that comprise immature stress fibers, which is particularly interesting in cells and tissues that contain regions of both well-formed and malformed z-lines. Other algorithms that isolate z-lines in an image[?] cannot provide this detailed biological information. As the actin co-stain is not always available, other algorithms have been developed to summarize sarcomere architecture from the z-line stain alone. We agree with the reviewer that our analysis adds to what has been done by others. For example, in Sutcliffe et al.[?], "sarcomere organization" describes local organization of α-actinin pixels by quantifying the correlation of pixel intensities within ~1-10 µm at different orientations, while the orientational order parameter (OOP) reported by `ZlineDetection` describes organization on a global, tissue

level (~40-100 µm)[?] . This distinction is particularly evident in how "sarcomere organization" and the OOP would describe an isotropic tissue, which is locally organized, but globally disorganized.
We thank the reviewer for their questions and believe we have greatly improved our manuscript by including a paragraph in the Discussion section that compares and contrasts
`ZlineDetection` with existing algorithms.

Pages 12-13, Lines 441-458

Existing algorithms that quantify sarcomere architecture from only a z-line stain, isolate z-lines from off-target staining by using signal processing to identify double wavelets in an image[?] , quantify local, micron-scale organization[?] , or manually remove off-target staining[?,?,?,?] . These previous works have used a range of metrics to then classify tissue or cell architecture, many of which describe similar properties to those provided by `ZlineDetection`. For example, the local sarcomere organization algorithm developed by Sutcliffe et al.[?] scored isolated cells without actin co-staining (Figure 4 and 6d in Sutcliffe et al.[?]) with a sarcomere organization index, which ranges from 0-2. The sarcomere organization index[?] was ~0.1 for a primary cell they qualitatively classified as "disorganized", but ~0.4 for both a "well-organized" primary cell and a reprogrammed cardiomyocyte, even though the latter had disorganized myofibrils and some α-actinin punctate patterns indicative of premyofibrils. By contrast, the α-actinin OOP, which measures organization globally, was 0.2, 0.52, 0.17 for the three cell types, respectively. As such, the cell-tissue scale OOP and the sarcomere scale organization index[?] provide qualitatively different measurements, but the key difference is in the scale at which the measurement is happening. Because tissue level organization influences the strength of tissue contraction[?] , we believe a global metric is essential.

> *The technique has been tested on cultured myocytes as well as primary myocytes, has this at all been tested in unstructured iPSC derived cardiomyocytes? Especially those that have not been cultured on a patterned surface. These would be cells that have a far higher variance in z-line structure. This could be of interest to a broad audience, due to the emergence of many models of disease in iPSC derived cardiomyocytes that display sarcomere malformations.*

Although we disrupted the z-line architecture of cells in two ways to test if `ZlineDetection` can be used to quantify immature morphology, we agree with the reviewer that it is also of interest to the community to test the performance on stem cell-derived cardiomyocytes. Therefore, we used `ZlineDetection` to analyze published images of stem cell-derived cardiomyocytes and have clarified the ability and utility of using `ZlineDetection` on stem cell-derived cardiomyocytes in the Discussion section.

Pages 13, Lines 458-464

Nevertheless, the main advantage of using the actin co-stain to isolate z-lines is the ability to quantify the amount of off-target staining based on the additional biological information. This can be especially useful in analyzing noisy stem cell-derived cardiomyocytes. For example, a square stem cell-derived cardiomyocyte shown in a previous publication[?] was easily analyzed with `ZlineDetection` (z-line OOP = 0.17, z-line fraction = 0.48, median continuous z-line length = 0.87 µm).

> *Is the algorithm reliant on the co-stain with actin or can it be run with just a z-disc label? Maybe this has already been stated somewhere. This could be a particular hurdle when looking at relatively unstructured iPSC derived cardiomyocytes.*

`ZlineDetection` will run with only a z-disc label, however without the actin co-stain `ZlineDetection` cannot distinguish between z-discs and punctate α-actinin indicative of premyofibrils. Therefore, without the actin co-stain, the metrics reported in this paper will summarize all α-actinin rather than only z-line architecture. A major advantage of `ZlineDetection` over existing algorithms is the ability to report on the z-line architecture and the myofibril formation separately, which requires the actin stain. Although

it is common to co-stain stem cell-derived cardiomyocytes with actin[?], we agree with the reviewer that it is of interest to the community, and the dependence of `ZlineDetection` on co-staining with actin was ambiguous. We appreciate the reviewer's question and have clarified in the Discussion section.

Page 12, Lines 438-441

In the absence of the actin co-stain, α-actinin pixels can no longer be classified as z-lines or off-target staining, and `ZlineDetection` can no longer decouple these two metrics and instead reports the simple α-actinin OOP that has been used previously[? ? ? ?].

> *Can the system be used on unfixed cells that have endogenous tags, or express fluorescent proteins for instance PMID 29364522 and 30956114.*

`ZlineDetection` can analyze unfixed cells that express fluorescent proteins, such as those published by Sharma et al.[?]. Analyzing such a video results in how the structure of the expressed protein changes over time. We analyzed a video published by Sharma et al.[?], and as expected, the orientational order was relatively constant over the course of a contraction. However, the median continuous z-line length varied due to the non-synchronous beating of the stem cell derived cardiomycoyte. We appreciate the reviewer's comment and recognize the growing availability and interest in producing and analyzing unfixed, labeled cells. We have included instructions for how to run `ZlineDetection` on videos of contracting myocytes into our user guide and have stated in the Discussion section the ability to analyze unfixed, labeled cells with `ZlineDetection`.

Page 13, Lines 508-513

It is worth noting that `ZlineDetetion` can be used with other stains or methods of visualizing cardiac striations. For example, it is possible to analyze videos previously published by Sharma et al.[?] of unfixed cells that express florescent proteins. Such an analysis in the absence of a co-stain would result in OOP of the expressed protein labeled structures and the striation lengths as a function of time (S6 Fig).

S6 Fig

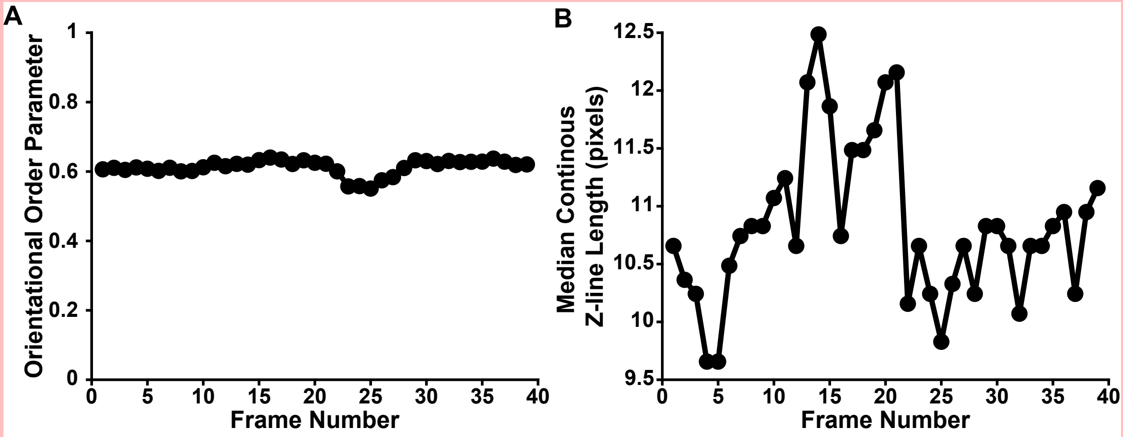

Analysis of beating cardiomyocyte. Results of analyzing titin-GFP sarcomere reporter human induced pluripotent stem cell-derived cardiomyocyte published by Sharma et al.[?]. **(A)** Orientational order parameter and **(B)** median continuous z-line length in pixels as a function of frame number. As expected, the OOP was relatively constant throughout the contraction, while the median continuous z-line length varied due to non-synchronous contractions of neighboring myofibrils.

> *When referencing z-line length, is there an explanation why the z-line length in figure 4 Iso and Aniso are so much lower than in figure 3i and 3ii? Is this borne out in the literature? Relatively speaking the error of the z-line measure in figure 3 has a far higher error, which is maybe surprising given that these are from patterned cells*

The z-line fraction of the anisotropic and isotropic tissues falls between the z-line fraction of single cells qualitatively classified as having “good” and those designated as having “intermediate” z-line architecture. This indicates that these tissues are comprised of both cells with good and cells with intermediate z-line architecture. It follows that the median continuous z-line length for the anisotropic and isotropic tissues is lower than the median continuous z-line length for “good” single cells and closer to that of cells with intermediate z-line architecture. We believe it is beneficial to explain how the results of analyzing tissues compares to those of single cells. We have included a detailed explanation into the Results section and are grateful to the reviewer for their question.

Page 11, Lines 385-392

While the z-line architecture of the single cells was expertly classified as good, intermediate, or bad, it was impractical to classify individual cells within whole tissues. Therefore, the z-line fraction of the anisotropic and isotropic tissues falling between the z-line fraction of good and intermediate single cells (Fig 3C) indicated that these tissues contain cells with both good and intermediate z-line architecture. Similarly, the median continuous z-line length for the anisotropic and isotropic tissues was lower than the median continuous z-line length for single cells with good z-line architecture and closer to that of cells with intermediate z-line architecture (Fig 3D).

> *Presumably the algorithm doesn't output sarcomere length for pairs of these z-lines in the resting stained cells (see PMID 30700234)? It could be of interest in many models of disease, drug application, and development to see if there is a correlate between resting sarcomere length and sarcomeric order.*

We thank the reviewer for this suggestion and have modified `ZlineDetection` to also output the the average sarcomere length. Because `ZlineDetection` outputs the location of all continuous z-lines, it is possible to look at the sarcomere length at each continuous z-line. We recognize that sarcomere length is a useful metric of cardiomyocyte maturity and have stated in the Discussion section that it can be reported by `ZlineDetection`

Page 13, Lines 464-466

While not an interesting metric in primary cardiomyocytes, `ZlineDetection` also reports the distance between z-lines (i.e. sarcomere length), which is often used to quantify cardiomyocyte maturity[?,?] and disease state[?].

> *Minor:*
> *What is the computing power needed to run this script. It would be useful to know how long a set of images take to run through the pipeline and if this can be done through a computing cluster if need be.*

We have only run `ZlineDetection` on personal computers, as it takes ~30 seconds to analyze a 1024 x 1344 image on a computer with 32GB of RAM. We agree that the computational requirements of an algorithm are important considerations and have included the computation power in the caption of S1 Fig. We appreciate the reviewer's suggestion and we expect knowing that `ZlineDetection` can be run on a personal computer will make it more accessible to the community.

> *Figure 3F the Y-axis label has been lost. In general the main text figures are very low resolution, making it hard to see the images clearly. The images in the supplement are of far higher resolution.*

Thank you for bringing this to our attention. We apologize for any confusion that we caused by using a small symbol in the y-axis label of Figure 3F. We have modified the figure and spelled out the axis name.

Figure 3

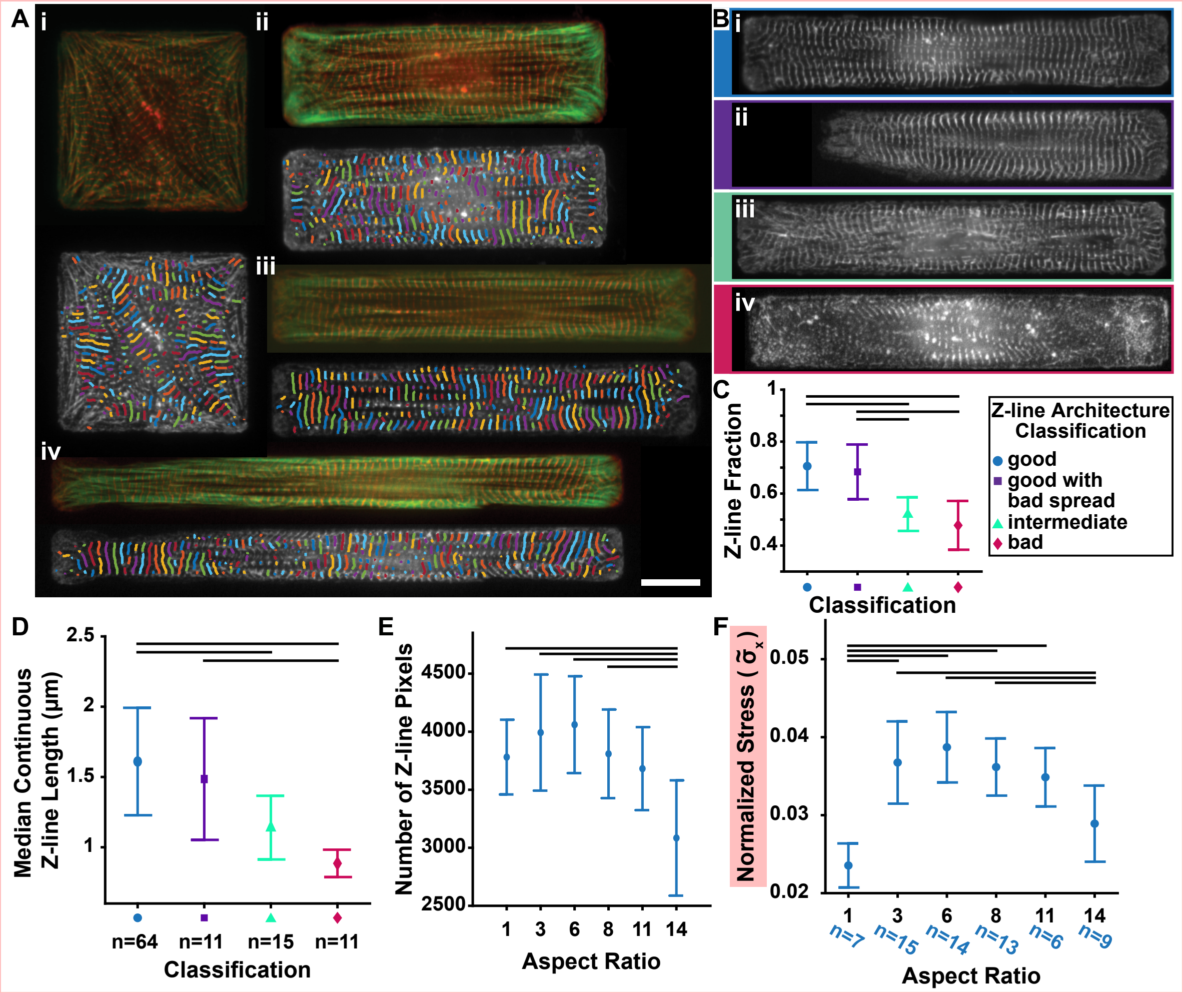

> *The Discussion / Conclusion section states that this system can aid in modelling force outputs, I think this statement may need to be reconsidered. Would this technique for instance predict changes in force, if the change in force were not accompanied by a change in z-line architecture? Such as could be hypothesized in hypertrophic or dilated cardiomyopathies.*

We are thankful to the reviewer and agree the original statement was too broad and thus overstated. We have rephrased that portion of the conclusion to clarify that it is changes to force production accompanied by changes to cytoskeletal architecture that our technique can assist in modeling.

Page 14, Lines 533-536

Having the ability to measure continuous z-lines can pave the way to predicting force measurements as it relates to z-line architecture in cardiac tissues through the use of experimental and mathematical modeling approaches.

> *Figure 4 bars for mean are not visible on my version, maybe this is due to a compression issue on submission. Please check.*

We apologize for any confusion the lower resolution image caused. The manuscript figures were compressed in the pdf of the manuscipt generated by the submission website, but the full resolution images are

linked and can be downloaded. We have included a higher resolution image where the bars for the mean are visible below.

Figure 4

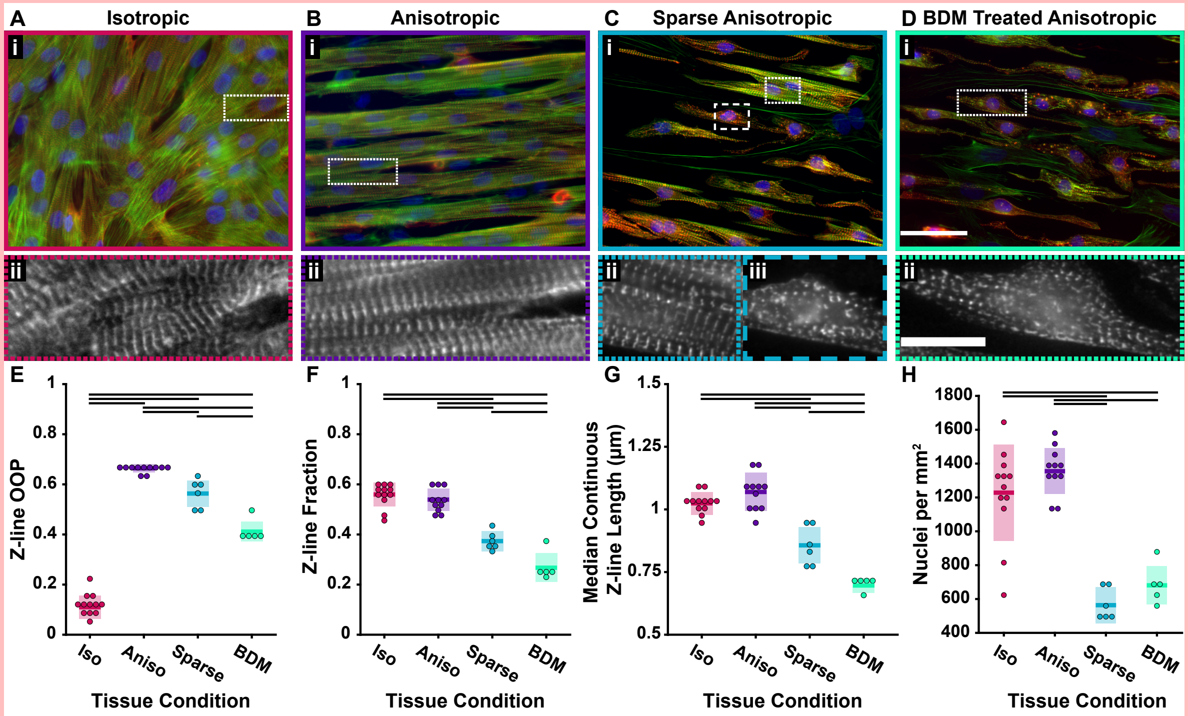

> *Figure 3 A you show the markups of the z-lines identified in the patterned cells, it would be nice to see how reliable this markup is in Figure 4 panels Aii, Bii, Cii and iii and Dii.*

We understand the benefit of seeing the plotted continuous z-lines on the tissue sections and thank the reviewer for this suggestion. There is not enough room in Figure 4 to include the plots at a high enough magnification, but we have included the plots as a supplemental figure (S4 Fig).

S4 Fig

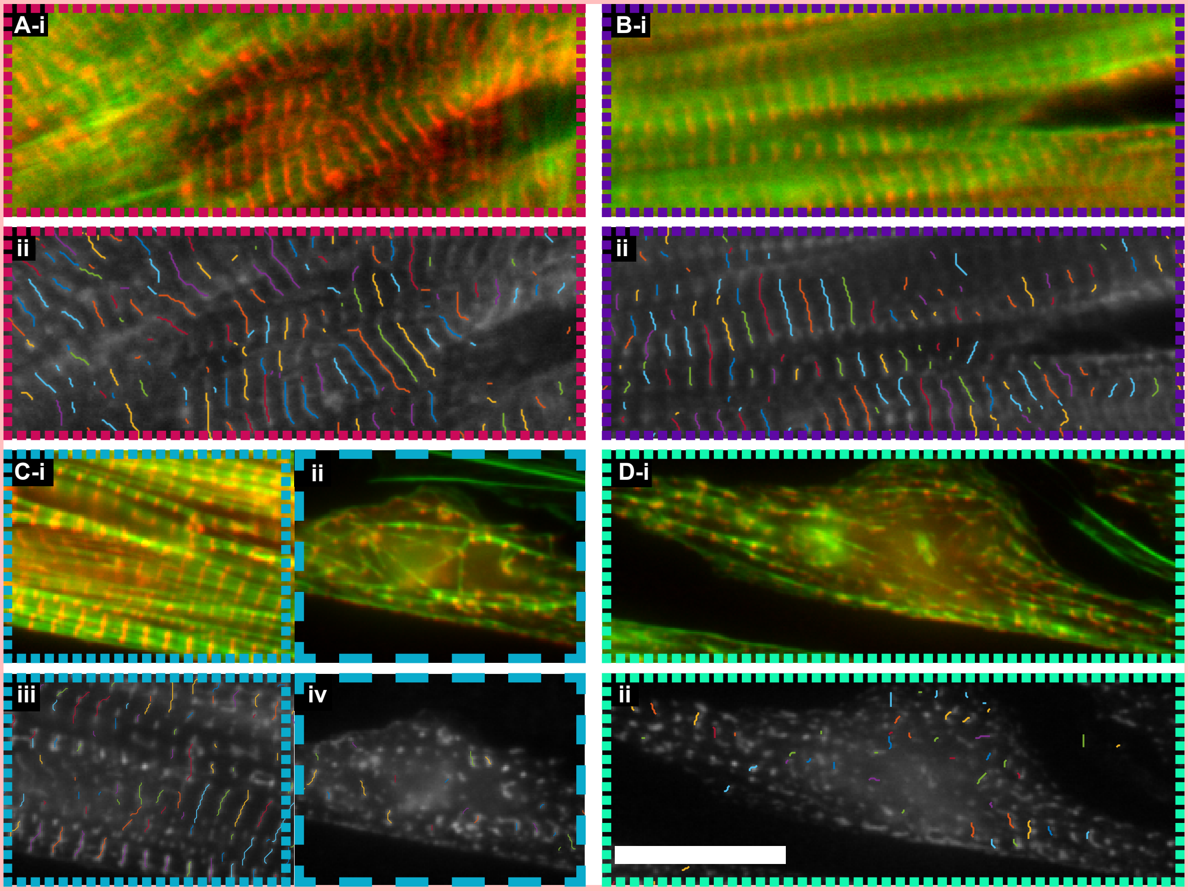

Continuous z-lines plotted on tissue segments. Sections of cardiac tissue shown in Figure 4 A-D stained for actin (green) and α-actinin (red) on a uniform layer of FN **(Ai)**, FN in lines **(Bi)**, FN in lines with sparsely seeded cardiomyocytes **(Ci)**, and FN in lines with cardiomyocytes treated with BDM **(Di)** and their corresponding continuous z-lines **(A-Dii)**. Scale bar: 15 µm

> *Z-line is sometimes capitalized in figures and sometimes lower case z-line.*

We thank the reviewer for making us aware of this issue. We have capitalized "z-line" in all figures.

References

[] Christopher N Toepfer, Arun Sharma, Marcelo Cicconet, Amanda C Garfinkel, Michael Mücke, Meraj Neyazi, Jon AL Willcox, Radhika Agarwal, Manuel Schmid, Jyoti Rao, et al. Sarctrack: an adaptable software tool for efficient large-scale analysis of sarcomere function in hipsc-cardiomyocytes. *Circulation research*, 124(8):1172–1183, 2019.

[] Matthew D Sutcliffe, Philip M Tan, Antonio Fernandez-Perez, Young-Jae Nam, Nikhil V Munshi, and Jeffrey J Saucerman. High content analysis identifies unique morphological features of reprogrammed cardiomyocytes. *Scientific reports*, 8(1):1258, 2018.

[] Nancy K Drew, Nicholas E Johnsen, Jason Q Core, and Anna Grosberg. Multiscale characterization of engineered cardiac tissue architecture. *Journal of biomechanical engineering*, 138(11):111003, 2016.

[] Po-Ling Kuo, Hyungsuk Lee, Mark-Anthony Bray, Nicholas A Geisse, Yen-Tsung Huang, William J Adams, Sean P Sheehy, and Kevin K Parker. Myocyte shape regulates lateral registry of sarcomeres and contractility. *The American journal of pathology*, 181(6):2030–2037, 2012.

[] Nancy K Drew, Mackenzie A Eagleson, Danny B Baldo Jr, Kevin Kit Parker, and Anna Grosberg. Metrics for assessing cytoskeletal orientational correlations and consistency. *PLoS computational biology*, 11(4):e1004190, 2015.

[] Anna Grosberg, Patrick W Alford, Megan L McCain, and Kevin Kit Parker. Ensembles of engineered cardiac tissues for physiological and pharmacological study: heart on a chip. *Lab on a chip*, 11(24):4165–4173, 2011.

[] Adam W Feinberg, Patrick W Alford, Hongwei Jin, Crystal M Ripplinger, Andreas A Werdich, Sean P Sheehy, Anna Grosberg, and Kevin Kit Parker. Controlling the contractile strength of engineered cardiac muscle by hierarchal tissue architecture. *Biomaterials*, 33(23):5732–5741, 2012.

[] Meghan B Knight, Nancy K Drew, Linda A McCarthy, and Anna Grosberg. Emergent global contractile force in cardiac tissues. *Biophysical journal*, 110(7):1615–1624, 2016.

[] Sean P Sheehy, Anna Grosberg, and Kevin Kit Parker. The contribution of cellular mechanotransduction to cardiomyocyte form and function. *Biomechanics and modeling in mechanobiology*, 11(8):1227–1239, 2012.

[] Arun Sharma, Christopher N Toepfer, Manuel Schmid, Amanda C Garfinkel, and Christine E Seidman. Differentiation and contractile analysis of gfp-sarcomere reporter hipsc-cardiomyocytes. *Current protocols in human genetics*, 96(1):21–12, 2018.

[] Scott D Lundy, Wei-Zhong Zhu, Michael Regnier, and Michael A Laflamme. Structural and functional maturation of cardiomyocytes derived from human pluripotent stem cells. *Stem cells and development*, 22(14):1991–2002, 2013.

[] Pieter P de Tombe and Henk EDJ ter Keurs. Cardiac muscle mechanics: sarcomere length matters. *Journal of molecular and cellular cardiology*, 91:148–150, 2016.

[] Nazha Hamdani, Viola Kooij, Sabine van Dijk, Daphne Merkus, Walter J Paulus, Cris dos Remedios, Dirk J Duncker, Ger JM Stienen, and Jolanda van der Velden. Sarcomeric dysfunction in heart failure. *Cardiovascular research*, 77(4):649–658, 2007.
